# Intraductal Carcinoma of the Parotid Gland as a Rare Neoplasm: A Case Report and Literature Review

**DOI:** 10.4314/ejhs.v33i1.21

**Published:** 2023-01

**Authors:** Eugénie Delaine, Andrea Avagnina, Stéphane Yerly, Sara Fontanella, Abderrahmane Hedjoudje, Bassel Hallak, Salim Bouayed, Asimakis D Asimakopoulos

**Affiliations:** 1 Department of Otorhinolaryngology, Hôpital de Sion, Centre Hospitalier du Valais Romand, Sion, Switzerland; 2 Cytopathology Laboratory, Institut Central des Hôpitaux, Hôpital du Valais, Sion, Switzerland; 3 Department of Radiology, Hôpital de Sion, Centre Hospitalier du Valais Romand, Sion, Switzerland

**Keywords:** Intraductal carcinoma, Low-Grade Salivary Duct Carcinoma, Intercalated duct carcinoma, Parotid carcinoma, Immunohistochemistry, Foci of invasion

## Abstract

**Background:**

Intraductal carcinoma is a rare low grade neoplasm of salivary glands with an excellent prognosis. It most frequently occurs in the parotid gland. Ectopic localizations are quite rare.

**Methods:**

This case report describes a man in his 60's who was referred to ear, nose and throat outpatient department with 1-month history of painless swelling of the right parotid region.

**Results:**

Ultrasound-guided fine-needle aspiration unveiled a cytologic specimen judged as “suspicious for malignancy” and patient underwent a partial superficial parotidectomy. Immunohistochemistry confirmed diagnosis of intraductal carcinoma of right parotid gland.

**Conclusions:**

There are few reported cases concerning this clinical entity following thorough review of the literature and recent developments with reference to the contribution of cytology and histopathology will probably modify its classification and management.

## Introduction

Most parotid tumors are benign with pleomorphic adenoma and Warthin tumors accounting for up to 94% of all neoplasms. Intraductal carcinoma (IDC) is a rare malignant tumor of the salivary gland recently reclassified by the World Health Organization (WHO) in 2017. In this article**,** we report a case of an IDC of the right parotid gland accompanied with a literature review.

## Case Presentation

A 63-year-old Caucasian male, smoker with multiple cardiovascular diseases was admitted to ear, nose and throat outpatient department with complaint of 1-month history of right cheek swelling.

On initial assessment he was apyretic and physical exam was notable for painless, non-erythematous edema of the right parotid region associated with a mobile and roundish mass, approximately 2cm in diameter.

**Figure 1:**
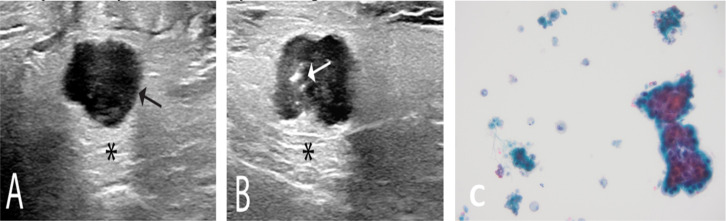
Ultrasonography of the right parotid gland and fine-needle aspiration cytology sample. Axial (A) and longitudinal (B) sonogram of the right parotid gland showing a single lobulated and well circumscribed hypoechogenic cystic lesion inside the superficial parenchyma (black arrow) with acoustic enhancement (black asterisk). The lesion present with few echogenic foci (white arrow) in the upper part that may represent debris or particles suspended in fluid. Liquid-based preparation of fine-needle aspiration of right intraparotid lesion (C) displaying pseudopapillary cluster in a cystic background with « dirty » necrosis and amorphous material (Papanicolau, 400x).

An ultrasonography was performed to determine differential diagnosis and identified a single lobulated and well circumscribed hypoechogenic cystic lesion with irregular edges, inside the superficial parenchyma of the gland (Figure 1A, B). Hence, an ultrasound-guided fine-needle aspiration (FNA) was performed revealing widespread “dirty” necrosis and cystic background with pseudo papillary clusters of moderately atypical cells with cyanophilic cytoplasm (Figure 1C).

Cytopathologic evaluation revealed a specimen classified in category V -suspicious for malignancy- according to Milan System for reporting salivary gland cytopathology, and diagnostic hypothesis of salivary duct carcinoma was considered.

**Figure 2:**
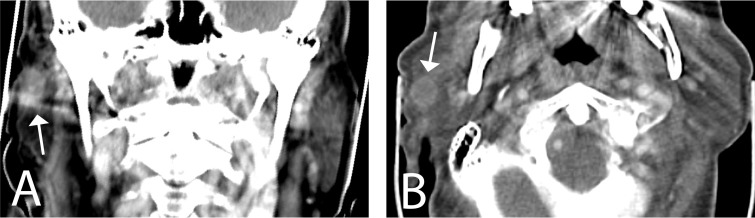
Representative face contrast-enhanced CT slices Coronal (A) and axial (B) reconstructions showing a single slightly heterogeneous, well circumscribed enhancing nodular lesion within superficial middle third (arrow) of right parotid gland characterized by a thin fibrous capsule surrounding the lesion.

Contrast-enhanced face and thoracic computed tomography (CT) demonstrated an homogeneously enhancing nodular lesion of the superficial lobe of right parotid gland after intravenous administration of a gadolinium-based contrast agent (Figure 2) and failed to reveal any suspicious cervical lymph node or synchronous thoracic lesion.

The patient underwent partial superficial parotidectomy under general anesthesia and intra-operative frozen section procedure confirmed a malignant epithelial tumor. Given its size and the absence of suspicious lymph node, a neck dissection was not performed.

**Figure 3:**
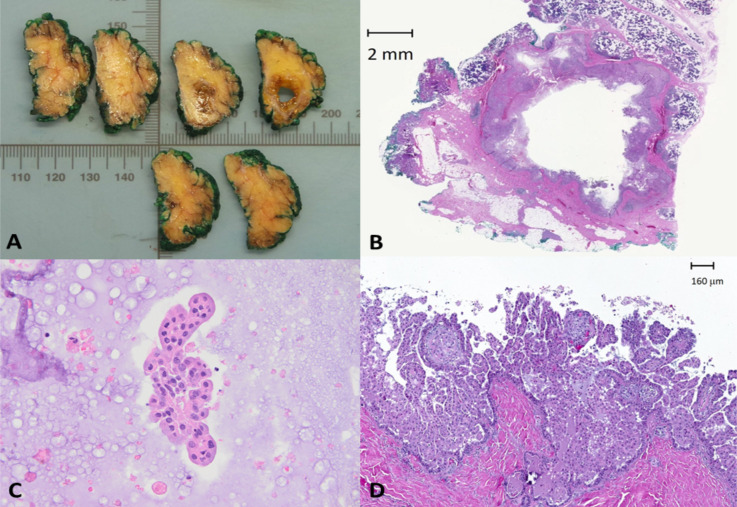
Cut section and cell-block preparation of the right parotid gland. (A= The tumor appeared as a cyst within the parotid gland; B=An histologic overview of the lesion (hematoxylin-eosin); C=Papillary cluster of moderately atypical cell with macrophages and necrotic cellular debris (hematoxylin-eosin, 400x); D=Microscopically, the wall of the cyst was lined by a proliferation with a papillary and micropapillary architecture (hematoxylin-eosin)

Grossly, the lesion consisted of a single cystic space, with a diameter of 1,3 cm (Figure 3A). Microscopically, the cyst had a smooth contour and was surrounded by a fibrous capsule (Figure 3B,D). The wall of the cyst was lined by a proliferation with a papillary and micropapillary architecture (Figure 3D). The cells had ovoid nuclei and abundant cytoplasm, and showed no apocrine features (Figure 3C).

By immunohistochemistry, a p63 positive myoepithelial cell layer was identified partially encircling the lesion. Microscopic foci of invasion, with no basal lining, were present outside of the capsule. There was no lymphovascular or perineural invasion and no lymph node metastasis. The lesion was completely excised.

The proliferation was positive for Cytokeratin 7, S100 and SOX10 by immunohistochemistry, and negative for DOG1, pNTRK and GATA3. A preliminary diagnosis of a low-grade encapsulate carcinoma, compatible with an intraductal, a secretory, or an acinic cell carcinoma was rendered, and the slides and blocks were sent for review and molecular analysis to an external institution.

The additional immunohistochemistry showed a focal positivity for mammmaglobin and negativity for androgen receptors.

A NCOA4-RET fusion, a rather specific chromosomal rearrangement, was identified in the lesion, permitting to confirm the diagnosis of intraductal carcinoma. The final diagnosis therefore, was of an intraductal carcinoma with foci of invasion.

## Discussion

IDC is a rare neoplasm of the salivary glands that most frequently occurs in the parotid gland. Chen in 1983 first reported a case of IDC of the minor salivary gland (1). It had been named Low-Grade Salivary Duct Carcinoma and Low Grade Cribriform Cystadenocarcinoma before being classified as Intraductal Carcinoma in the latest edition of the WHO classification of Head and Neck tumors in 2017 (2).

Even if the WHO definition of the tumor is an «intracystic/intraductal proliferation of neoplastic epithelial cells », molecularly confirmed cases of invasive IDC are now described, to the point that some investigators propose to rename this entity as « intercalated duct carcinoma » (2). Moreover, a recent study defined the dogma that IDC is an in-situ neoplasm by demonstrating that the myoepithelial cells harbour the same rearrangement as the ductal cells, consequently they are neoplastic as well (3).

A variety of testing approaches have been employed and new molecular testings have been rapidly developed with regard to IDC. There is no agreement about the number of tumor subtypes, but at least four have been described (intercalated, apocrine, oncocytic and mixed) (2). It largely affects adults with a wide age spectrum (range 27–93, mean 61,4) and it has a female predilection with a ratio 1,5/1 (4).

Histologic differential diagnosis may be challenging and includes cystadenoma, variants of adenocarcinoma not otherwise specified, including cystadenocarcinoma, sclerosing polycystic adenosis/adenoma, and papillary cystic variant of acinic cell carcinoma and (mammary analogue) secretory carcinoma.

Patients typically present with an asymptomatic and slow growing parotid mass, usually not particularly aggressive, without invasion of the surrounding tissues and facial nerve involvement (4).

Salivary gland tumors are radiologically assessed using ultrasonography and magnetic resonance imaging. Ultrasound-guided FNA is the modality of choice for a quick and cheap initial evaluation of these neoplasms. Most FNA cases of IDC would be signalized by uncertain malignant potential on the basis of cytomorphology according to the current Milan System for Reporting Salivary Gland cytopathology, due to the wide range of morphological features, variety of IDC subtypes and limitation of cytology, and thus the role of molecular analysis is expected to be essential (5).

Concerning its management, previous reports recommended surgical excision with negative margins (4). Giovacchini et al. also reported that radiation therapy or chemotherapy are not indicated in the absence of lymph node or distant metastasis (4). In advanced and metastatic cases, molecular changes including RET-rearranged tumors could represent potential therapeutic targets (5).

IDC is a rare but widely debated neoplasm, with excellent prognosis, and its management consists of exclusive complete surgical excision. Our knowledge is based on isolated case reports and small-sized retrospective studies which reflect a high level of heterogeneity. Owing to its rarity, implicated practitioners encounter difficulties to differentiate it from other more invasive, but with similar morphological features, neoplasms of salivary glands. Recent advancements in cytology and histopathology are expected to reshape its categorization and management. That way, proper diagnosis will lessen invasive and adjuvant treatments.
